# The Glymphatic System in Traumatic Brain Injury, Subarachnoid Hemorrhage, and Neurodegeneration: A Unifying Neurobiological Framework

**DOI:** 10.3390/biomedicines14102314

**Published:** 2026-10-12

**Authors:** Naeem Ul Haq, Sardar Umer Irshad, Zainab Bibi, Sawera Rehman, Sumra Sohail, Sardar Remsha Irshad, Noor-ul-Eman Haider, Shah Mir, Asad Shareef, Brandon Lucke-Wold

**Affiliations:** 1 Department of Neurosurgery, Mardan Medical Complex, Mardan 23200, Pakistan; 2 Bacha Khan Medical College, Khyber Medical University, Mardan 23200, Pakistan; sardarumer606@gmail.com (S.U.I.); drzainabbibi0@gmail.com (Z.B.); sumra05533@gmail.com (S.S.); 3 Khyber Girls Medical College, Khyber Medical University, Peshawar 25100, Pakistan; sawerarehman960@gmail.com (S.R.); remshasardar0@gmail.com (S.R.I.); 4 Khyber Medical College, Khyber Medical University, Peshawar 25120, Pakistan; noorulemanhaider@gmail.com (N.-u.-E.H.); dr.shahmir073@gmail.com (S.M.); 5 Department of Pharmacy, Quaid-i-Azam University Islamabad, Islamabad 45320, Pakistan; asadsk986@gmail.com; 6 Department of Neurosurgery, University of Arizona, Tucson, AZ 85721, USA

**Keywords:** glymphatic system, aquaporin-4 (AQP4), meningeal lymphatics, traumatic brain injury, subarachnoid hemorrhage, neurodegeneration, cerebrospinal fluid clearance, sleep, DTI-ALPS, chronic traumatic encephalopathy

## Abstract

Whether glymphatic dysfunction drives or merely follows brain injury and neurodegeneration remains unresolved. The glymphatic system mediates the transport, exchange, and clearance of solutes between cerebrospinal fluid (CSF) and interstitial fluid (ISF) through paravascular CSF influx, polarized aquaporin-4 (AQP4) at astrocytic endfeet, and meningeal lymphatic drainage. This narrative review synthesizes evidence on glymphatic regulation and its role in traumatic brain injury (TBI), subarachnoid hemorrhage (SAH), and neurodegeneration, and appraises imaging, biomarker, and therapeutic approaches. PubMed/MEDLINE and Google Scholar were searched from inception to August 2026, and 128 sources were analyzed. In TBI, mechanical shear and NLR family pyrin domain-containing 3 (NLRP3)-dependent inflammation may alter AQP4 distribution and impair tau and amyloid-beta (Aβ) clearance; however, glymphatic failure has not been established as the causal mediator of later neurodegeneration, which is likely multifactorial. After SAH, blood and fibrin may obstruct perivascular pathways, and hemoglobin, heme, and iron toxicity may further disrupt CSF–ISF exchange; these changes are associated with delayed cerebral ischemia, cortical spreading depolarizations, and post-hemorrhagic hydrocephalus. Impaired clearance of Aβ, phosphorylated tau, and α-synuclein may link glymphatic dysfunction to Alzheimer’s and Parkinson’s diseases, although causality is unproven. Current imaging tools, including diffusion tensor imaging analysis along the perivascular space (DTI-ALPS), and biomarkers do not yet provide a validated measure of glymphatic function. Sleep optimization is a low-risk strategy, and controlled lumbar drainage has the strongest clinical evidence among CSF-dynamics approaches. Translation requires appropriate animal models and prospective TBI/SAH studies combining serial DTI-ALPS with cognitive follow-up.

## 1. Introduction

The brain parenchyma lacks histologically identifiable lymphatic vessels, and protein waste was traditionally thought to be removed by intracellular degradation, such as autophagy and ubiquitination, with only a few proteins, such as amyloid-β (Aβ), also cleared by specific transport across the blood–brain barrier (BBB). Interstitial solutes were assumed to reach the cerebrospinal fluid (CSF) by diffuse bulk flow of interstitial fluid (ISF) rather than through a discrete structure [1,2]. Using in vivo two-photon imaging, Iliff et al. showed that CSF enters the parenchyma along paravascular spaces surrounding penetrating arteries and that ISF is cleared along paravenous pathways, and they named this glial water flux-dependent pathway the “glymphatic” system [1]. CSF then crosses the astroglial endfeet, mixes with ISF, and ultimately exits along perineural sheaths, meningeal lymphatic vessels, and arachnoid granulations, although the relative importance of these routes is still debated [2]. Serial T1-weighted magnetic resonance imaging (MRI) after intrathecal gadobutrol showed comparable CSF transport in the human brain [2].

Astrocytic endfeet ensheathe the cerebral vasculature, and the water channel aquaporin-4 (AQP4) is highly polarized to these perivascular endfeet, where it is proposed to provide a low-resistance route for fluid movement into the interstitium [3]. In AQP4-null mice, paravascular tracer movement into the parenchyma was markedly reduced, and clearance of [3H] mannitol and 125I-Aβ1–40 fell by about 70% and 55%, respectively [1]. A later report of normal CSF clearance in AQP4-knockout animals questioned this role [2,3], but multiple independent groups replicated the original findings [2], and a meta-analysis showed significantly reduced tracer transport in knockout animals, with anesthesia, age, and tracer delivery explaining the discordant results [3]. A critical review nevertheless concluded that solute influx and efflux occur along periarterial routes, that evidence for perivenous outflow is lacking, that interstitial flow is too slow to rival diffusion, and that AQP4 probably does not act as a water conduit between perivascular and interstitial spaces, its precise role remaining unclear [4].

Meningeal lymphatic vessels extended this framework beyond the parenchyma. Louveau et al. identified functional vessels along the dural sinuses that expressed lymphatic vessel endothelial hyaluronan receptor 1 (LYVE-1), prospero homeobox protein 1 (PROX1), podoplanin, and vascular endothelial growth factor receptor 3 (VEGFR3) and carried fluid and immune cells from the CSF to the deep cervical lymph nodes (dCLNs) [5]. Aspelund et al. independently described a dural network draining through skull-base foramina, in which aplasia impaired clearance of intraparenchymal ovalbumin to the dCLNs while paravascular outflow remained detectable, suggesting that meningeal lymphatics act downstream of the glymphatic pathway [6]. Together, these findings indicate a two-tiered architecture of AQP4-dependent paravascular transport and meningeal drainage [1,5,6].

Glymphatic efficiency is further shaped by physiological state. Reducing arterial pulsatility by unilateral carotid ligation diminished paravascular tracer influx, whereas increasing it with dobutamine accelerated influx, although pulsatility is only one of several contributing factors [7]. Natural sleep and anesthesia were associated with an approximately 60% expansion of the cortical interstitial space, increased CSF–ISF exchange, and roughly twofold greater Aβ clearance [8], while body posture also matters, with Aβ clearance greater in the lateral than in the supine or prone position [9]. An intrinsic circadian rhythm adds further modulation: glymphatic influx and clearance peaked in the mid-rest phase regardless of anesthetic and paralleled perivascular AQP4 polarization, whereas lymphatic drainage to the dCLNs followed an opposing rhythm [10]. In humans, electroencephalography (EEG) and functional MRI (fMRI) showed that non-rapid eye movement (NREM) sleep couples slow-delta EEG activity, blood-oxygen-level-dependent (BOLD) signals, and CSF inflow, with EEG amplitude peaking about 6.4 s before the CSF flow peak [11]. Aging illustrates how these regulators converge: in aged mice, CSF–ISF exchange, Aβ clearance (about 40% lower), and arterial pulsatility (27% lower) declined alongside loss of perivascular AQP4 polarization, which may increase vulnerability to neurotoxic solute accumulation [12]. Because AQP4 polarization, arterial pulsatility, sleep, and meningeal drainage all influence clearance, disturbance of any of them could plausibly compromise waste removal.

Although these mechanisms have been characterized largely under physiological conditions, it remains unresolved whether glymphatic dysfunction is a driver or merely a consequence of brain injury and neurodegeneration. The aim of this review is, therefore, to synthesize current evidence on the role of the glymphatic system in traumatic brain injury (TBI), subarachnoid hemorrhage (SAH), and neurodegeneration and to evaluate its potential as a diagnostic and therapeutic target. After outlining the molecular and physiological determinants of glymphatic function, we examine how injury alters AQP4 polarization and perivascular and meningeal lymphatic clearance; how clearance failure relates to delayed cerebral ischemia, cortical spreading depolarizations, and post-hemorrhagic hydrocephalus; its links with Alzheimer’s and Parkinson’s disease; and the imaging, biomarker, and therapeutic approaches that follow from it.

### Methodology

This narrative review is based on a literature search of PubMed/MEDLINE and Google Scholar from inception to August 2026. Search terms included “glymphatic,” “aquaporin-4,” “meningeal lymphatics,” “traumatic brain injury,” “subarachnoid hemorrhage,” “neurodegeneration,” “sleep,” and “DTI-ALPS,” used in various combinations. We included English-language original studies, reviews, and meta-analyses relevant to glymphatic physiology and its role in TBI, SAH, and neurodegeneration. Reference lists of key articles were also screened for additional sources. Studies were selected by the authors based on relevance, and priority was given to human and clinical evidence, systematic reviews, and well-designed experimental studies, with seminal preclinical work included where it established core mechanisms. As this is a narrative review, no formal risk-of-bias assessment was performed. In total, 128 sources were included, comprising 84 original studies (including 3 randomized controlled trials), 40 reviews (37 narrative reviews and 3 systematic reviews or meta-analyses), 3 consensus or position statements, and 1 clinical scale.

## 2. Molecular and Cellular Machinery of Glymphatic Function

### 2.1. Aquaporin-4 Polarization and Its Regulation

In astrocytes, water homeostasis across the blood–brain and blood–spinal cord barriers is supported by AQP4, a specialized water channel protein. AQP4 is present in higher concentrations at perivascular astrocytic endfeet, where it supports glymphatic clearance of extracellular solutes such as Aβ [13,14,15]. This perivascular enrichment is established primarily by structural mechanisms that govern AQP4 assembly and anchoring, and it is further modulated by signaling pathways that regulate AQP4 abundance at the plasma membrane, particularly under pathological conditions.

First, polarization of AQP4 at astrocytic endfeet depends on its assembly into orthogonal arrays of particles (OAPs). The M23 isoform is responsible for the formation of larger OAPs, while the A25Q mutation disrupts these structures into tetramers. Although total AQP4 levels remained unchanged, its localization at astrocytic endfeet was decreased, which protected mice from brain edema after water intoxication and middle cerebral artery occlusion (MCAO)/reperfusion [14]. The AQP4 readthrough isoform (AQP4X), generated by C-terminal stop-codon readthrough, promotes the polarization of AQP4 and α-syntrophin around blood vessels, and disrupting this readthrough altered the clearance of contrast agents from the brain on MRI [15].

Second, polarized AQP4 is anchored at perivascular endfeet through its interaction with α-syntrophin, a component of the dystrophin-associated protein complex [16]. Loss of α-syntrophin displaces AQP4 from perivascular and subpial endfeet without changing total AQP4 expression; the anchoring machinery, including the role of the dystroglycan complex, is described in Section 2.2.

In addition to these structural determinants, AQP4 abundance at the astrocyte plasma membrane is dynamically regulated, and this regulation is engaged under pathological conditions. Hypoxia and hypotonicity, which are encountered in edema and injury, trigger transient receptor potential vanilloid 4 (TRPV4)-dependent calcium influx, which activates calmodulin (CaM) and, subsequently, protein kinase A (PKA). PKA phosphorylates AQP4 at Ser276, while CaM binds its carboxyl terminus and induces a conformational change, together promoting AQP4 translocation to the plasma membrane and increasing membrane water permeability [13]. Accordingly, inhibiting CaM (trifluoperazine) or PKA (H89) prevented AQP4 relocation to the blood–spinal cord barrier, reduced brain and spinal cord edema after injury, and improved recovery in rats [13]. These findings indicate that hypoxia and hypotonicity act as context-dependent modulators of AQP4 membrane localization during injury, rather than as determinants of its baseline polarization.

### 2.2. Astrocytic Endfoot Structure and Dystroglycan Complex Anchoring

Evidence for this α-syntrophin-dependent anchoring comes from knockout studies. Mice lacking α-syntrophin (α-Syn−/−) had a marked loss of AQP4 from perivascular and subpial astrocytic endfeet membranes despite showing normal total brain AQP4 levels. In contrast, AQP4 levels at the neuropil-facing membrane remained preserved and even increased [16]. This mislocalization of AQP4 was associated with swollen perivascular endfeet under normal conditions, indicating reduced water efflux. Subsequent to the transient occlusion of the middle cerebral artery, mice showed partial protection from postischemic edema and infarction. However, ischemia also affected AQP4 localization in normal mice, where perivascular AQP4 was lost after reperfusion even though α-syntrophin was present [16].

The dystroglycan complex also helps organize AQP4 into clusters through its interaction with perivascular laminin. Laminin treatment in cultured astrocytes induces the clustering of AQP4 together with β-dystroglycan, dystrophin, utrophin, and syntrophin in GM1-positive lipid raft macrodomains. The formation of these clusters depends on intact cholesterol-rich lipid rafts and dystroglycan, as cholesterol disruption and dystroglycan silencing (siDag1) decreased cluster number and size [17]. The laminin–dystroglycan–AQP4 assay was used to screen over 3500 compounds, and six inhibitors of clustering were identified. Chloranil, a quinone compound, was more potent than flunarizine and inhibited clustering by inducing metalloproteinase-dependent shedding of β-dystroglycan, which severed laminin binding to the dystroglycan complex. In primary rat astrocytes, this shedding was blocked by prinomastat, a matrix metalloproteinase inhibitor with selectivity for MMP-2, -9, -13, and -14 and by tissue inhibitors of metalloproteinases (TIMP)-2 and TIMP-3 but not TIMP-1, implicating matrix metalloproteinase-2 (MMP-2) [18]. Shedding was also prevented by co-incubation with N-acetylcysteine (NAC), an antioxidant and scavenger of reactive oxygen species (ROS), indicating that the effect of chloranil depends on ROS generation upstream of metalloproteinase-mediated cleavage [18]. This finding is consistent with the well-recognized antioxidant properties of NAC, which in this setting served as an experimental tool to establish ROS dependence rather than as a neuroprotective intervention. Because NAC is a thiol-containing compound, it may also interact directly with the electrophilic quinone. These in vitro observations should, therefore, not be taken to imply that antioxidant treatment preserves AQP4 polarization in vivo.

### 2.3. Meningeal Lymphatic Vessels (Dural Sinuses)

Meningeal lymphatic vessels provide a complementary route for the clearance of CSF and interstitial solutes from the central nervous system (CNS). Louveau et al. reported that these vessels have punctate junctions and a discontinuous basement membrane, characteristic of initial lymphatic vessels, and that they carry intracerebroventricularly administered QDot655 and drain Evans blue into the dCLNs. Resection of the dCLNs increased meningeal T-cell accumulation [5]. In another study, Aspelund et al. described a network of dural lymphatic vessels located alongside the superior sagittal and transverse sinuses. These vessels extended through basal foramina alongside blood vessels and cranial nerves. The lymphatic vessel endothelium expressed LYVE-1, podoplanin, and C-C motif chemokine ligand 21 (CCL21) [6].

Absinta et al. demonstrated the presence of meningeal lymphatic vessels in primates by using gadobutrol-enhanced T2 fluid-attenuated inversion recovery (FLAIR) and black-blood MRI [19]. These vessels followed a course that was along the dural sinuses and showed enhancement distinct from that produced by gadofosveset. Their lymphatic identity was further confirmed by immunohistochemical analysis, which showed these vessels expressed lymphatic markers including LYVE-1, PROX1, podoplanin, chicken ovalbumin upstream promoter transcription factor II (COUP-TFII), and CCL21 [19].

### 2.4. Neurovascular Coupling and Arterial Pulsatility as the Clearance “Pump”

Using in vivo two-photon line scanning, Iliff et al. found that arterial wall pulsatility was greatest in penetrating arteries compared with surface arteries and veins. Reducing pulsatility by about 50% through unilateral carotid ligation slowed paravascular CSF tracer influx, whereas increasing it by about 60% with dobutamine enhanced influx, indicating that arterial pulsatility directly influences paravascular CSF transport [7]. Particle tracking velocimetry showed that CSF movement along periarterial spaces is pulsatile at cardiac frequency and that arterial wall speed matches CSF speed. These results provide support for perivascular pumping as the driving mechanism [20]. Acute hypertension did not change arterial diameter but modified the pattern of arterial wall pulsation. This increased backflow and reduced net perivascular space (PVS) flow speed by about 40% [20]. In contrast to the proposed role of arterial pulsations, Kedarasetti et al. used computational fluid-dynamic simulations to examine their role in PVS flow. They reported that 1–4% arterial diameter pulsations produced large oscillatory movements, whereas only a small pressure difference of ~0.01 mmHg was required to produce directional CSF movement [21].

Van Veluw et al. found that spontaneous low-frequency (~0.1 Hz) arteriolar diameter fluctuations contribute to paravascular dextran clearance in awake mice. Experimental increase in vasomotion through visual stimulation resulted in enhanced dextran clearance, whereas defective vascular reactivity in amyloid precursor protein/presenilin 1 (APP/PS1) mice was associated with reduced clearance [22]. Arterial pulsation is, therefore, best regarded as one of several contributors to perivascular transport, alongside slower vasomotion and the resistance of the interstitial space, rather than as its sole driver; how these factors change during sleep is discussed in Section 2.5.

### 2.5. Circadian and Sleep-Stage Dependence (Norepinephrine Tone, Interstitial Volume Changes)

In addition to arterial pulsations, glymphatic clearance is associated with the sleep–wake cycle and its underlying neurochemical and circadian regulators. These factors can modulate how efficiently the glymphatic system clears substances from the brain. In the study by Xie et al. cited above, pharmacological inhibition of adrenergic receptors in awake mice produced an expansion of the interstitial space similar to those observed during sleep [8]. The inhibition also increased CSF tracer influx toward levels recorded during sleep or anesthesia. This treatment implies that noradrenergic tone plays an important role in regulating interstitial volume [8].

The recent literature states that during NREM sleep, infraslow norepinephrine oscillations from the locus coeruleus induce slow and large-amplitude vasomotion, which is associated with glymphatic clearance. In contrast, sleep-promoting agents such as zolpidem do not reproduce these noradrenergic oscillations, and therefore, do not enhance glymphatic flow in the same manner [23]. Under three different anesthetics, a mid-day peak was observed in glymphatic influx that was independent of the external light–dark cycle. The rhythm was observed to persist under constant light for 10 days and was lost in AQP4-deficient animals along with the opposing rhythm in cervical lymph node drainage [10].

Taken together with Section 2.4, these findings indicate that sleep-related enhancement of glymphatic exchange does not contradict a role for arterial pulsation. During sleep, reduced noradrenergic tone expands the interstitial space, which lowers resistance to CSF–ISF exchange and increases tracer influx and clearance [8]. In NREM sleep, infraslow norepinephrine oscillations additionally generate slow, large-amplitude vasomotion [23], a process that is also modulated by neural activity through neurovascular coupling [22]. Because cardiac-frequency pulsation produces mainly oscillatory CSF movement, with net directional flow depending on small pressure differences [21], these changes in interstitial resistance and vasomotion may allow the same arterial driving forces to move more fluid and solute during sleep.

**Figure 1:**
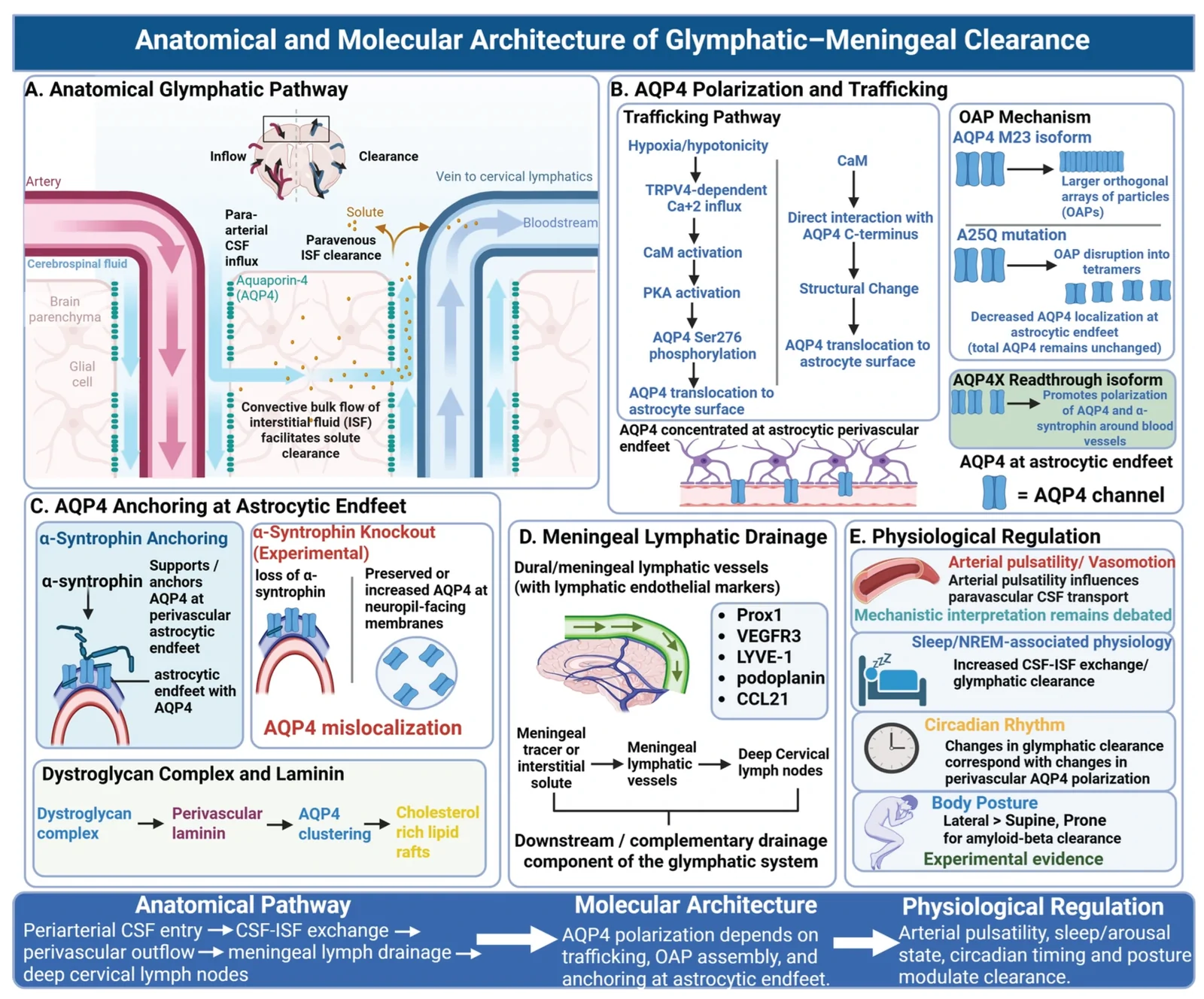
Anatomical and molecular architecture of glymphatic–meningeal clearance. (**A**) Periarterial CSF influx, ISF exchange, and paravenous outflow. (**B**) Orthogonal array of particles (OAP) assembly and AQP4 trafficking. (**C**) Astrocytic endfoot anchoring of AQP4 via α-syntrophin and the dystroglycan complex. (**D**) Meningeal lymphatic drainage to the deep cervical lymph nodes. (**E**) Physiological regulators of clearance, including arterial pulsatility, NREM sleep, circadian rhythm, and body posture. **Abbreviations:** A25Q, alanine-to-glutamine substitution at residue 25 of AQP4; AQP4, aquaporin-4; AQP4X, AQP4 readthrough isoform; Ca^2+^, calcium ion; CaM, calmodulin; CCL21, C-C motif chemokine ligand 21; CSF, cerebrospinal fluid; ISF, interstitial fluid; LYVE-1, lymphatic vessel endothelial hyaluronan receptor 1; M23, AQP4 M23 isoform; NREM, non-rapid eye movement; OAP, orthogonal array of particles; PKA, protein kinase A; PROX1, prospero homeobox protein 1; Ser276, serine residue 276; TRPV4, transient receptor potential vanilloid 4; VEGFR3, vascular endothelial growth factor receptor 3.

Fultz et al. demonstrated that CSF inflow at the fourth ventricle undergoes large, coherent oscillations at approximately 0.05 Hz during human NREM sleep. These oscillations were anticorrelated with cortical BOLD signals and were generated by slow-delta EEG amplitude fluctuations [11]. Consistent with this, Eide et al. reported that total sleep deprivation impaired the clearance of an intrathecal tracer from various brain regions. A subsequent night of recovery sleep did not restore clearance levels to normal [24]. These findings and observations collectively suggest that glymphatic flow is regulated by norepinephrine-driven vasomotion, interstitial volume dynamics, and circadian timing. These mechanisms together influence the efficiency and timing of glymphatic clearance. These anatomical, molecular, and physiological determinants of glymphatic–meningeal clearance are summarized in Figure 1.

### 2.6. Additional Molecular and Barrier Components Beyond AQP4

AQP4 receives the most attention in this review because it is the best-characterized molecular determinant of glymphatic solute transport [3]. However, AQP4 operates within the neurovascular unit, and other proteins also shape perivascular fluid and solute handling and the barrier properties of the vessel wall.

Dystrophin is a component of the dystrophin-associated protein complex discussed in Section 2.2, which anchors AQP4 at perivascular endfeet through α-syntrophin [16]. In dystrophin-deficient (mdx) mice, brain AQP4 protein declines progressively with age (about 30% at 1 month and 70% at 1 year), with marked loss at swollen perivascular astrocytic endfeet, whereas AQP4 mRNA is unchanged [25]. In aged mdx mice (18–20 months), the brain also shows open endothelial tight junctions, reduced ZO-1 protein, diffuse rather than banded claudin-1 staining, and increased vascular permeability [26]. These findings associate dystrophin deficiency with reduced perivascular AQP4 and impaired barrier integrity, although whether glial or endothelial changes occur first remains unresolved [26].

LRP1 contributes to Aβ clearance through more than one route. At the BBB, it is described as the primary receptor mediating Aβ transport from brain to blood, one of several clearance mechanisms alongside ISF bulk flow, glymphatic paravascular clearance, CSF absorption, and enzymatic degradation [27]. LRP1 is also expressed in astrocytes: its knockdown in primary astrocytes reduced Aβ uptake and degradation and lowered IDE, MMP2, and MMP9 mRNA, while astrocyte-specific Lrp1 deletion in APP/PS1 mice slightly but significantly prolonged ISF Aβ40 half-life and increased Aβ levels and plaque deposition without altering APP processing [28]. The same study also notes that AQP4 deletion has been reported to suppress astrocytic LRP1 near plaques, whereas astrocytic LRP1 deletion did not significantly change AQP4 levels, suggesting AQP4 may act upstream of LRP1, though cooperative function cannot be excluded [28].

Tight junctions between brain capillary endothelial cells severely restrict paracellular entry of circulating molecules into the brain [29]. In a rat controlled cortical impact model, occludin was reduced from 1 to 14 days after injury and claudin-5 at 1 and 3 days, with ZO-1 and endothelial markers also reduced at 24 h, and BBB permeability showed an early and a delayed phase [29]. In a mouse cold-injury model of focal cortical edema, transient siRNA-mediated suppression of claudin-5 produced a size-selective BBB opening to molecules up to approximately 1 kDa for 24–72 h and was associated with lower tissue water content, smaller lesion volume, and improved neurological and cognitive outcome; the authors proposed that this allowed excess water to move from brain to blood [30]. These studies measured barrier permeability, tissue water content, and outcome rather than CSF–ISF exchange, so the contribution of claudin-5 and occludin to glymphatic transport itself remains to be tested.

## 3. Glymphatic Dysfunction in Traumatic Brain Injury

Traumatic brain injury (TBI) is defined as an alteration in brain function, or other evidence of brain pathology, caused by an external force [31]. Severity has conventionally been graded using the total Glasgow Coma Scale (GCS) score as mild (13–15), moderate (9–12), or severe (≤8), although the 2024 NINDS Clinical/Symptoms Working Group considers this system unsatisfactory because it overlooks variation in injury severity, clinical needs, and prognosis [32]. The GCS itself was introduced in 1974 to assess the depth and duration of impaired consciousness through three independently evaluated components: eye opening, verbal response, and motor response [33]. Sport-related concussion is defined as a traumatic brain injury induced by biomechanical forces; however, the term concussion is imprecise, is often used interchangeably with mild TBI, and it remains unresolved whether concussion lies on a TBI spectrum with lesser degrees of diffuse structural change or reflects reversible physiological changes [34]. Experimental and clinical studies suggest that TBI can disrupt brain homeostasis through alterations in astrocytic AQP4 organization, inducing cerebral edema and axonal damage, which may disrupt perivascular pathways and reduce glymphatic transport [35]. TBI not only causes mechanical and hemodynamic disturbance, but also leads to slowed and desynchronized brain oscillatory activity, which impairs glymphatic function. Disruptions in gamma and beta oscillatory activity correlate with deficits in working memory, attention and neuroplasticity, causing neurodegenerative disorders [36].

### 3.1. Mechanical Disruption of AQP4 Polarity Post-Impact

As described in Section 2, glymphatic clearance depends on polarized AQP4 at astrocytic endfeet and on downstream meningeal lymphatic drainage [3,37,38,39], and AQP4 polarity is increasingly recognized as a determinant of brain health and resilience to neurodegeneration. TBI-induced mechanical shear forces and pressure waves trigger immediate astrocyte activation and loss of AQP4 polarization [13]. Although the precise sequence in TBI is not fully defined, several mechanisms described in Section 2 are plausible. AQP4 polarization depends on anchoring through α-syntrophin and the dystroglycan–laminin complex [16,17], and metalloproteinase-mediated shedding of β-dystroglycan, which depends on ROS, can sever this anchoring in vitro [18]. Injury-related hypoxia and hypotonicity may additionally drive TRPV4–calmodulin–PKA-dependent relocation of AQP4 to the plasma membrane [13], and NLRP3–caspase signaling has been associated with loss of perivascular AQP4 polarization [40]. Whether altered expression or redistribution of connexin 43 contributes to this process was not addressed in the sources reviewed here, and it remains an open question for future studies.

These secondary processes alter the normal function of astrocytes as well as the localization and expression of the AQP4 channel [41,42]. This causes astrocyte swelling and the delocalization of AQP4 following TBI. It also causes the breakdown of the normal structure and glymphatic impairment as well as the formation of cytotoxic edema [41,42]. Experimental studies have highlighted the dual role of AQP4 in TBI. Due to ionic imbalances and BBB disruption, AQP4 contributes to early cytotoxic edema by allowing excessive water influx into astrocytes. This leads to the swelling of brain tissue, increased intracranial pressure (ICP), and further neuronal injury [43]. In later stages, AQP4 facilitates vasogenic edema clearance by enhancing glymphatic drainage and CSF circulation. It also helps to reduce swelling and improve recovery. In mild TBI (mTBI) and moderate TBI models, the perivascular polarization of AQP4 is disrupted at the endfeet of reactive astrocytes [44]. Furthermore, the genetic knockout of AQP4 aggravates post-TBI glymphatic dysfunction. This leads to an accumulation of tau protein, S100 calcium-binding protein B (S100B), glial fibrillary acidic protein (GFAP), and neuron-specific enolase (NSE) [44]. Pyroptosis-related neuroinflammation is increasingly regarded as a contributor to secondary injury after TBI. Activation of the NLRP3–caspase pathway has been associated with loss of perivascular AQP4 polarization and increased blood–brain barrier permeability, which may further impede the clearance of metabolic waste [40].

Secondary injury after the primary mechanical insult involves excitotoxicity, mitochondrial dysfunction, oxidative stress, inflammation, blood–brain barrier (BBB) disruption, and cell death [45,46]. Mitochondrial injury increases ROS production and impairs cellular energy production [47], and the resulting oxidative stress causes lipid peroxidation and oxidation of proteins and DNA [45,47]. Together, oxidative stress and mitochondrial dysfunction promote apoptotic cell death [47]. Because astrocytes are central to the glymphatic system, these processes may plausibly contribute to astrocyte reactivity and AQP4 depolarization after TBI. However, direct evidence connecting oxidative-stress- or apoptosis-specific pathways to glymphatic failure after TBI is currently limited.

Nitric oxide (NO) [48] and hydrogen sulfide (H_2_S) [49,50] are gaseous signaling molecules studied in brain injury, but their role in glymphatic regulation after TBI was not examined in the studies reviewed here. NO depletion occurs early after brain injury, including TBI, alongside reduced blood flow, and its effects depend on the source: eNOS-derived NO appears protective, whereas iNOS-derived NO may contribute to injury [48]. In a phase II trial in TBI patients, the iNOS-preferring inhibitor VAS203 improved extended Glasgow outcome scores versus placebo, although experimental iNOS data after TBI are conflicting [48]. For AQP4, in rats with middle cerebral artery occlusion and reperfusion, AQP4 mRNA decreased in the cortex and striatum, and pretreatment with the NOS inhibitor L-NAME prevented this decrease in the striatum only [51]. In a rat TBI model, pretreatment with the H_2_S donor sodium hydrosulfide (NaHS) improved motor and memory performance and reduced brain water content, apoptosis, glutamate, and oxidative-stress markers, but AQP4 was not assessed [50]. In a rat SAH model, NaHS pretreatment reduced the increase in astrocytic AQP4 protein together with reduced microglial activation, proinflammatory cytokines, and brain edema [49]. In all three experimental studies, the agent was given before injury, none assessed perivascular AQP4 polarization or CSF tracer clearance, and the AQP4 data derive from ischemia and SAH rather than TBI [49,50,51]. These studies therefore do not establish whether NO or H_2_S regulate AQP4 polarization and glymphatic clearance after TBI.

Clinical studies generally point toward consistent upregulation of AQP4 after TBI, which is often linked to edema development and neuroinflammation. In contrast, experimental studies have shown heterogeneous or even contradictory patterns of AQP4 expression depending on the model, species, and time point [52]. Furthermore, some studies emphasized disrupted AQP4 polarity as a driver of edema severity rather than absolute expression [53].

### 3.2. Animal Models: Closed-Head Injury, Tracer Clearance Studies

Animal models provide clear evidence that TBI changes glymphatic fluid movement and clearance. In rodent models of TBI, a 60% reduction in perivascular CSF influx is evidence of glymphatic dysfunction [54]. Using contrast-enhanced MRI (CE-MRI), one study demonstrated mTBI-induced dysfunction in both CSF influx and efflux. Glymphatic dysfunction in post-injury rodents emerges as early as 1 day, with the effect persisting at least up to 1 month [55].

TBI in mice leads to decreased glymphatic influx and impaired clearance of intracortically injected radiotracers from the brain interstitium persisting for more than 28 days after the injury. Several disorders are known in which abnormal ISF dynamics is caused due to mechanical force [54]. A model of moderate to severe trauma in mice demonstrated that the migration of tracer injected into the perivascular cortex after injury was significantly reduced compared with the contralateral region to trauma, suggesting reduced activity of the glymphatic system, and this finding was continued 28 days after the injury [54]. Impaired glymphatic function in AQP4 knockout mice increases the accumulation of phosphorylated tau and axonal degeneration after TBI [56]. These processes lead to cognitive decline in mice. Thus, levels of GFAP, S100B and NSE were elevated in blood serum at 18 h after TBI in wild-type mice, but inhibition of glymphatic efflux prevented the rise in these serum biomarkers [56].

Another animal experiment using CE-MRI indicated that repetitive mTBI caused an increased fluid influx to the brain tissue but reduced efflux throughout the limbic structures and olfactory bulb, but neither the influx nor efflux was altered in the cortical structures [57]. Researchers observed chronic impairment of glymphatic function in a murine model of repetitive blast mTBI. They also observed the emergence of sleep–wake disruption [42]. Importantly, glymphatic efflux in murine brains may transport biomarkers of TBI to the general circulation [56]. The observed sleep disturbances resembled daytime sleepiness and nighttime insomnia typical in post-TBI humans. Similar to diminished AQP4 polarization, post-TBI sleep deprivation can reduce glymphatic flow [42].

If a similar glymphatic efflux of potential biomarkers occurs in humans after TBI, some of the interventions commonly applied in clinical practice, e.g., sleep deprivation due to frequent neurological examination and ventriculostomy after severe TBI, seem likely to inhibit glymphatic function, thus potentially interfering in biomarker efflux, which could contribute to the difficulties in identifying a TBI blood test [58].

### 3.3. Human Evidence: DTI-ALPS Index Changes Post-TBI

New and emerging imaging techniques are being developed to study glymphatic dysfunction after TBI. Some techniques include using diffusion tensor imaging (DTI). DTI can be used to calculate the analysis along the perivascular space (ALPS) index, referred to as DTI-ALPS [59]. This method is proposed as an indirect index of glymphatic function in various neurologic diseases, including TBI. DTI-ALPS-based findings post-subacute TBI support a correlation between disease severity based on blood levels of neurofilament light chain (NfL), a highly sensitive TBI biomarker, and glymphatic system disruption [38]. Initial correlational analyses reveal an inverse relationship between diminished ALPS indices and elevated CSF Aβ/tau concentrations. This suggests that ALPS may reflect glymphatic clearance capacity, although it has not been validated as a biomarker (see Section 6 and Section 8) [60].

An increased amount of enlarged high-convexity perivascular space was detected on T2-weighted images during the observation of MRI in human. This finding might be a marker of potential inflammatory changes associated with mTBI [61]. The enlarged high-convexity perivascular space probably reflects early and permanent brain changes, which might reflect the accumulation of inflammatory cells and changes in vascular permeability [61]. Complementary DCE–MRI techniques quantitatively characterize glymphatic kinetics through contrast agent retention/clearance profiles, with methodology validation established in stroke models [62].

Human studies have reported both increases and decreases in glymphatic function after TBI. These discordant results likely originate from fundamental differences between studies, including patient populations, types and timing of injury, and imaging methodologies [63]. Some authors have proposed mechanistic explanations, suggesting that reduced post-mTBI glymphatic activity may result from loss of AQP4 polarization, whereas increased activity could represent a compensatory response to facilitate waste removal [63]. Even though existing clinical reports of accumulating evidence are currently few in number, such proof-of concept studies lay an important groundwork for future multicenter clinical trials [40]. Combining our preclinical mechanistic insights with the growing body of clinical biomarkers will continue to form the basis upon which we may provide disease-targeted interventions [40].

### 3.4. Temporal Course: Acute Failure vs. Chronic Impairment

In rodent models, glymphatic dysfunction has been detected as early as 1 day after TBI (the earliest time point examined) and persists for at least one month (Section 3.2) [54,55]. Altered AQP4 expression has also been reported within 72 h of injury [52,53].

During the acute phase, reactive astrogliosis primarily exerts protective effects. Activated astrocytes form the perilesional glial barrier, which sequesters the core injury area and confines the spread of damage and infiltration of inflammatory cells, thereby preserving surrounding healthy tissue. Astrocytes also contribute to the formation and regulation of cerebral edema through specific ion channels like AQP4 [13].

During the chronic phase, the function of astrocytes is more contradictory. They maintain the activation and stabilization of the glial scar through long-term activity. This forms an obstacle to axonal regeneration and the recovery of neural circuits. At the same time, it also has an impact on recovery in some active ways, including secretion of various neurotrophic factors [13]. Sleep fragmentation is highly prevalent after TBI. It is associated with impaired clearance of neurotoxic metabolites, persistent neuroinflammation, and cognitive decline [64]. These findings support a potential bidirectional relationship between TBI, sleep disruption and glymphatic clearance [65].

In some models, AQP4 expressions decrease globally, whereas in other models, AQP4 expression is upregulated. Another contrast lies in regional and temporal specificity. The clinical studies typically detect systemic or persistent AQP4 increases (e.g., in CSF, plasma), whereas experimental data often reveal biphasic patterns, regional variation, or opposite trends in core vs. peripheral brain zones [66]. While clinical studies largely support the pathological role of AQP4 overexpression in TBI, experimental data reveal a more complex and dynamic picture, with model-specific variations in AQP4 expression, localization, and regulation [66].

Chronic or repeated TBI was also found to affect the brain’s cognitive and executive functions as well as its structure. Several studies have pointed to how TBI and its subsequent effects on the glymphatic system lead to cognitive dysfunction that is commonly progressive in nature, and this has been proposed as one possible contributor to delayed cognitive dysfunction in diseases such as chronic traumatic encephalopathy (CTE) and other forms of dementia [67].

### 3.5. Link to Post-Traumatic Tau/Amyloid Accumulation and CTE Risk

Waste clearance failure may contribute to TBI pathophysiology. It involves cellular damage due to an impact to the head and release of harmful molecules to the brain interstitium [68]. Experimental and clinical studies suggest that injury-induced edema, axonal damage, and astroglial reactivity may disrupt perivascular pathways. These pathways can also be altered by cerebral small-vessel disease, white matter hyperintensities, and chronic hypertension. These changes in vascular pulsatility and BBB integrity compromise glymphatic flow [35]. Compromised glymphatic system clearance has been associated with toxic protein accumulation, including Aβ and tau. This accumulation in turn potentially leads to the development of Alzheimer’s disease [69].

Accumulation of metabolic byproducts after TBI has been proposed as one contributor to long-term neurodegenerative consequences [60]. Impaired fluid dynamics is coupled with elevated Aβ and phosphorylated tau levels in both CSF and interstitial compartments. This impairment not only acutely compromises neuronal synaptic function but also induces sustained neuroinflammatory responses and glial activation. These changes may then increase the risk of developing AD-like pathology or CTE [60].

TBI is also a known risk for late-life neurodegeneration. In CTE, an abnormal accumulation of Aβ and transactive response DNA-binding protein 43 (TDP-43) is observed, and in recent years, tauopathy has been indicated. Recent studies suggest that dysfunction in the glymphatic system may contribute to the development of dementia following TBI [59].

Glymphatic dysfunction is unlikely to act in isolation. Post-traumatic neurodegeneration is multifactorial. Axonal injury [69], neuroinflammation, vascular injury and blood–brain barrier dysfunction [35], mitochondrial injury, and tau-related pathology may each contribute independently or interact with impaired clearance. Glymphatic impairment may, therefore, represent one contributor, a consequence, or a marker of these processes, and the available data do not allow its relative contribution to be determined. The proposed sequence of TBI, glymphatic dysfunction, impaired tau/Aβ clearance, protein accumulation, and CTE- or Alzheimer’s disease-like pathology should accordingly be regarded as a plausible hypothesis rather than an established pathway.

**Figure 2:**
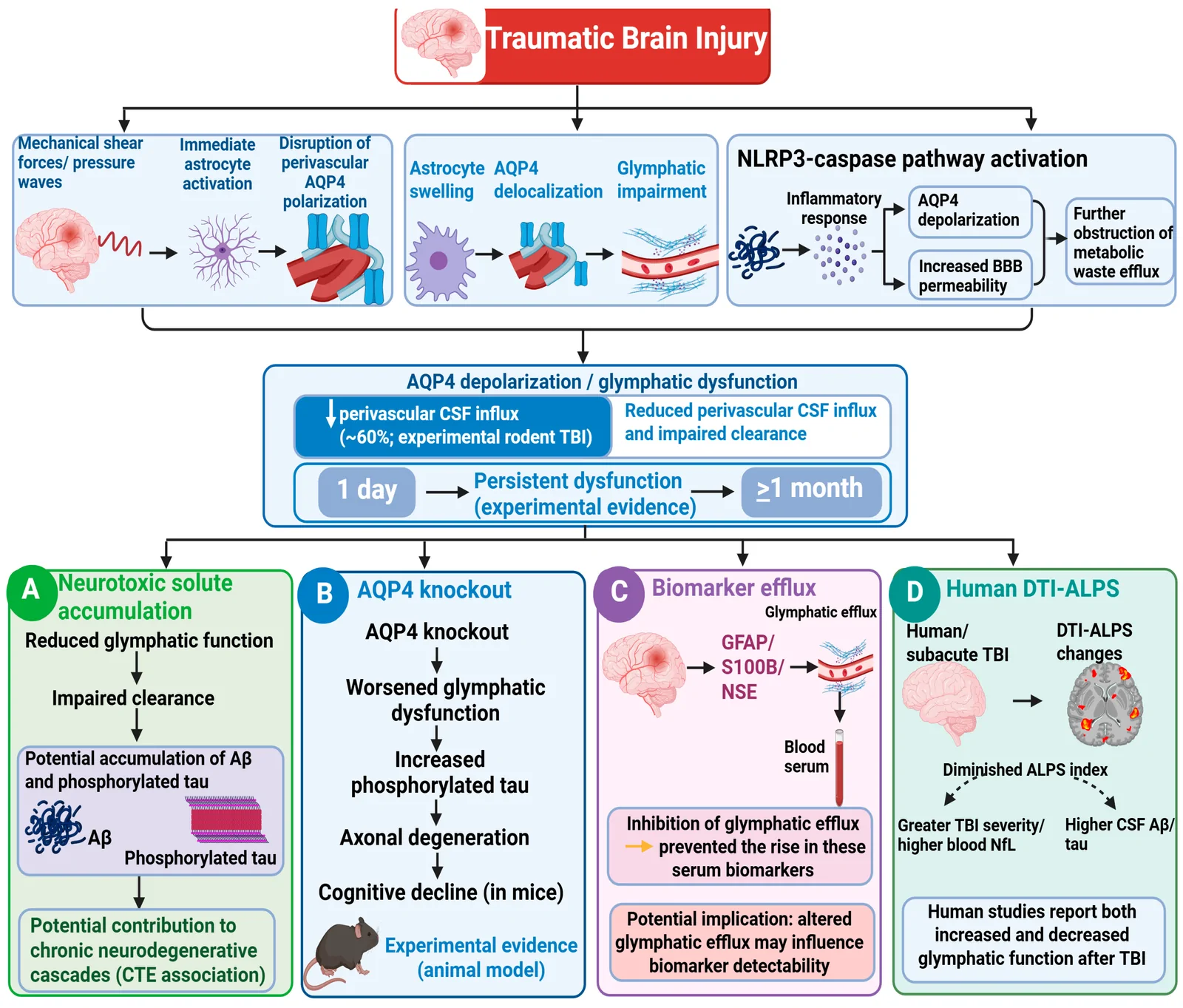
Glymphatic dysfunction in TBI. Mechanical shear forces and NLRP3–caspase pathway activation cause AQP4 depolarization and glymphatic impairment (reported from 1 day and persisting ≥ 1 month in experimental models), leading to (**A**) neurotoxic solute accumulation and potential contribution to CTE, (**B**) worsened glymphatic dysfunction and cognitive decline in AQP4-knockout mice, (**C**) serum biomarker efflux, and (**D**) DTI-ALPS changes in humans. **Abbreviations:** ALPS, analysis along the perivascular space; AQP4, aquaporin-4; Aβ, amyloid-β; BBB, blood–brain barrier; CSF, cerebrospinal fluid; CTE, chronic traumatic encephalopathy; DTI-ALPS, diffusion tensor imaging analysis along the perivascular space; GFAP, glial fibrillary acidic protein; NfL, neurofilament light chain; NLRP3, NLR family pyrin domain-containing 3; NSE, neuron-specific enolase; S100B, S100 calcium-binding protein B; TBI, traumatic brain injury.

The emerging studies linking TBI, glymphatic disruption, sleep and risk of neurodegenerative disorders support a complex dynamic association between specific trauma-induced physiological perturbation and chronic neuropathological changes [60]. However, the available evidence does not establish glymphatic failure as the causal mediator of post-traumatic neurodegeneration. Glymphatic-related measures such as DTI-ALPS have been associated with injury severity [59], but they have not been validated as diagnostic or prognostic biomarkers (see Section 6 and Section 8). Figure 2 summarizes the converging mechanical, inflammatory, and molecular pathways linking TBI-induced glymphatic dysfunction to biomarker release and neurodegenerative risk.

## 4. Glymphatic Dysfunction in Subarachnoid Hemorrhage

SAH, often resulting from aneurysmal rupture, remains a life-threatening cerebrovascular disorder with high morbidity and mortality. SAH induces a cascade of pathological changes that critically impair the function of the glymphatic system [70]. Following SAH, acute ICP elevation, subarachnoid blood clot burden, loss of arterial pulsatility, venous congestion, astrocytic AQP4 perivascular depolarization, and neuroinflammation occurs. These changes suppress the CSF-ISF exchange and outflow in the glymphatic system [71]. Persistent clearance failure promotes the retention of blood breakdown products, inflammatory mediators and metabolic waste. This amplifies microvascular dysfunction, cortical spreading depolarizations (SDs), BBB disruption, and secondary ischemic injury [70,71].

### 4.1. Blood Breakdown Products (Hemoglobin, Iron) as Glymphatic Obstruction/Toxicity

#### 4.1.1. Mechanical Obstruction and Blood Entry

SAH is characteristically described as blood that influxes into the subarachnoid space, mixes with the CSF throughout the entire circulation system of brain CSF, and diffuses throughout the brain [72]. Immediately after aneurysm rupture, extravasated blood rapidly fills the subarachnoid space, particularly the basal cisterns, and extends into periarterial spaces. This causes the physical obstruction of CSF conduits and acutely suppresses periarterial CSF influx into the brain parenchyma [71]. This obstruction coincides with a sudden ICP surge, which may further compress perivascular spaces and collapse glymphatic conduits [70,71].

In animal models, erythrocytes are found within subarachnoid, ventricular and perivascular spaces shortly after SAH. Intact erythrocytes are also found in the perivascular space of postmortem SAH patients [71]. Decreased glymphatic fluid exchange has similarly been observed in rodents and non-human primates, with impaired tracer distribution from the cisterna magna to perivascular spaces and parenchyma [73]. Fibrin/fibrinogen deposits occupy the perivascular space and inhibit CSF–ISF exchange, and fibrinogen in perivascular spaces suggests clot-mediated occlusion of glymphatic CSF pathways [71,74].

#### 4.1.2. Blood Toxicity and Astrocytic Dysfunction

Extravasated blood in the subarachnoid space further compounds this dysfunction. Hemolysis releases free hemoglobin, heme, and iron, which are potent pro-oxidants that amplify ROS generation, while coagulation releases thrombin and fibrinogen, which exert proinflammatory and neurotoxic effects [70]. These blood-derived components are detrimental not only to tissue adjacent to the vasculature but also to the parenchyma, where they trigger astrocyte reactivity and AQP4 depolarization, impairing the directional water transport required for effective CSF–ISF exchange [70]. The disrupted BBB intensifies local cytokine release (e.g., interleukin-1β [IL-1β], tumor necrosis factor-α [TNF-α]), creating a positive feedback loop of inflammation that impairs vascular compliance and reduces arterial pulsatility [71]. Iron released from erythrocyte degradation activates microglia and choroid plexus macrophages, facilitating proinflammatory cytokine release and oxidative stress [75]. Iron accumulation and oxidative stress also raise the possibility that programmed forms of cell death, particularly ferroptosis, contribute to glymphatic impairment after SAH. Because hemolysis releases free iron and potent pro-oxidants that amplify ROS generation [70], iron-dependent oxidative injury to astrocytes could plausibly contribute to AQP4 depolarization. However, none of the studies reviewed here directly tested ferroptosis in relation to glymphatic function, so this remains hypothetical. The same reasoning may apply to TBI, where oxidative stress, lipid peroxidation, and apoptotic cell death occur, but direct evidence connecting them to glymphatic failure is limited (Section 3.1). Thus, mechanical obstruction, blood toxicity, BBB disruption, and astrocytic dysfunction converge to impair glymphatic clearance after SAH [70,71].

### 4.2. Impaired CSF Outflow and Its Relationship to Delayed Cerebral Ischemia and Vasospasm

Delayed cerebral ischemia (DCI) is currently understood as a multifactorial process that evolves over time. The first 24 to 48 h after SAH are referred to as the early brain injury phase. It is largely characterized by the sequelae of increased ICP and transient global ischemia. During this period, cerebral edema, BBB disruption, sympathetic nervous system activation, autoregulatory failure, microthrombosis, SDs, and inflammation have all been observed [76]. The loss of cerebral autoregulation has been associated with an increase in microvascular spasms. It has been correlated with the development of DCI [77]. A recent clinical study further demonstrated that an increased risk of vasospasm is associated with impaired sphenoparietal venous drainage [71]. Oxyhemoglobin and other blood-breakdown products in the CSF induce endothelial dysfunction, inflammation, and vasoconstriction, which, in turn, can lead to brain ischemia and infarction [78].

DCI, occurring in 20–30% of SAH patients at 4–14 days after onset, is a major predictor of poor outcomes. One of the most immediate consequences of SAH is a sudden and dramatic elevation in ICP, which compresses the perivascular spaces essential for CSF influx [70]. This mechanical disruption collapses the arterial perivascular channels through which CSF normally enters the brain parenchyma, thereby halting the convective bulk flow that drives glymphatic clearance [70].

Animal studies have shown that persistent glymphatic dysfunction impairs CSF–ISF exchange, resulting in neurotoxic waste accumulation, sustained inflammation, and vascular disruption. This contributes to microthrombi formation, microvasospasm, and ICP spikes after SAH [70]. Glymphatic impairment is greater in the hemisphere ipsilateral to blood injection in animal models, consistent with the clinical pattern of DCI in SAH patients. This suggests that regional glymphatic dysfunction may contribute to its spatial development and distribution [70]. In rabbit models of SAH, vasospasm occurs not only in the basilar artery but also in the deep cerebral veins. These clinical observations showing elevated basal vein flow velocity correlated with preserved neurological function. The reduced flow velocity predicted persistent deficits in SAH patients [71]. However, the pathological components of DCI are intimately interconnected, with vascular dysfunction, inflammation, and SDs contributing to the disease process [71].

### 4.3. Brief Connecting Notes to Cortical Spreading Depolarizations

SDs are slowly propagating waves of almost complete membrane depolarizations in both neuronal and glial cells. SDs are initiated by similar mechanisms in metabolically compromised brain tissue but can be more severe and long lasting. Passive diffusion of extracellular K^+^ and glutamate provoke depolarization in surrounding gray matter. A positive feedback loop mediated by N-methyl-D-aspartate (NMDA) receptor- and Ca^2+^ channel-dependent glutamate release contributes to the self-sustaining nature of SDs. K^+^ cations and free hemoglobin in the subarachnoid space are characteristics of hemolyzed red blood cells after SAH [79]. The relationship between these processes is further supported by the accumulation of blood breakdown products within the perivascular and parenchymal compartments. Persistent glymphatic dysfunction promotes retention of blood breakdown products and inflammatory mediators, amplifying microvascular dysfunction and SDs [71].

NO is directly involved in this NMDA receptor- and Ca^2+^-dependent process. NMDA receptors and voltage-gated Ca^2+^ channels mediate Ca^2+^ elevations during SDs and are directly modulated by NO [80]. In rat and human neocortical slices, NO synthase (NOS) inhibition lowered the SD threshold; in rat slices, this effect was abolished by P/Q-type Ca^2+^ channel or NMDA receptor blockade but not by guanylate cyclase inhibition [80]. The threshold was maintained mainly by endothelial NOS and depended on basal NO rather than the NO surge during SDs [80]. Because intracellular Ca^2+^ stimulates NOS, NOS activity is likely increased during SDs [81]. In addition, NMDA receptor-mediated Ca^2+^ influx activates neuronal NOS (nNOS) through postsynaptic density protein-95 (PSD-95), and disrupting this coupling reduced excitotoxic and ischemic injury, although in stroke rather than SD models [82].

After SAH, hemoglobin released by hemolysis acts essentially as a NO scavenger [81], and elevated extracellular K^+^ and reduced NO have been reported [80]. In rats, raising K^+^ together with hemoglobin or a NOS inhibitor converted spreading hyperemia into spreading ischemia, whereas raising K^+^ alone did not [81]. Since glymphatic dysfunction promotes retention of blood breakdown products and amplifies SDs [71], impaired clearance may prolong exposure to NO-scavenging hemoglobin and K^+^ and lower the SD threshold. This remains hypothetical, as none of the cited studies measured glymphatic function.

SDs evoke a hyperemic response in healthy tissues as a result of the increased metabolic demand during disrupted ion homeostasis. Injured or compromised brain tissues are more prone to neurovascular uncoupling and often show hypoperfusion after SDs [83]. Due to BBB dysfunction in the intermediate phase, neurovascular coupling is compromised, and cerebral vasospasm is likely to occur [84]. Neurovascular uncoupling is characterized by neuronal hyperexcitability, dysregulated vascular contractility, and decreased metabolic waste clearance. This reduces the neuronal energy supply and leads to apoptosis [85].

A landmark study found that SDs occur after SAH in humans and predict the development of DCI. Later studies revealed that clusters of SDs magnify the duration of tissue hypoxia. Clustered SDs may be more important to DCI pathology than isolated depolarizations. The correlation between SDs and DCI remains even after the successful treatment of angiographic vasospasm. These findings support the interdependency between pathological factors after SAH [85]. The relationship between glymphatic dysfunction and SDs remains interconnected with the vascular and inflammatory components of SAH. Persistent glymphatic dysfunction contributes to microthrombi formation and microvasospasm. The sustained microvasospasm triggers SDs and may ultimately promote DCI [70]. The scientific rationale for targeting SDs to reduce DCI is strong, although powerful clinical trials are required to demonstrate a clear benefit in patients [85].

### 4.4. Hydrocephalus as a Clinical Manifestation of Glymphatic/CSF Outflow Failure

Hydrocephalus is a common and devastating complication of SAH occurring during both the acute and chronic phases and contributing substantially to long-term neurological morbidity and cognitive decline [70]. Hydrocephalus occurs in roughly 20–30% of SAH patients (the literature ranges from 6% up to 67%). It typically develops in the acute phase (within 3 days of hemorrhage) or subacute phase (4–14 days), while chronic hydrocephalus can manifest later in 10–20% of cases [86]. Persistent glymphatic dysfunction after SAH promotes CSF stagnation and impairs ISF clearance. This increased resistance to ventricular outflow leads to ventricular enlargement [70]. Acutely, blood clots can obstruct CSF pathways (e.g., blocking the fourth ventricular outlets or basal cisterns), leading to acute obstructive hydrocephalus. In the subacute and chronic periods, hemoglobin breakdown products trigger inflammation and fibrosis in the arachnoid granulations, which impairs CSF reabsorption. Inflammatory cascades involving transforming growth factor-β, MMPs, iron, and other mediators ultimately produce scarring. This results in the impaired CSF outflow, resulting in a communicating hydrocephalus [87].

In a beagle model of SAH induced by endovascular perforation, the temporal pattern was investigated up to 2 weeks using contrast-enhanced MRI. SAH caused an early and severe reduction in glymphatic function, detectable within hours, which persisted for 1 week and partially recovered at 2 weeks [88]. The impaired contrast gadolinium diethylenetriamine penta-acetic acid (Gd-DTPA) influx and efflux was detectable within 1 h, being most pronounced on the ipsilateral side of the brain [70]. This coincides with acute clot formation, transient arterial injury and early CSF pressure elevation. The progressive hydrocephalus and neurological deficits that peak at day 1 and partially recover by 1–2 weeks implicate sustained glymphatic collapse in post-SAH hydrocephalus and delayed dysfunction [71].

Temporary CSF diversion is a cornerstone of hydrocephalus management. External ventricular drainage (EVD) relieves ICP. The lumbar drainage can, alternatively, be used in communicating hydrocephalus [89]. The combination of EVD and lumbar drainage has been proposed to enhance clearance of subarachnoid blood and decrease vasospasm risk [89]. Cisternostomy has also been proposed to reduce swelling and wash away harmful substances, although its methodological limitations require better-controlled studies [90]. The mismatch between experimental and clinical temporal resolution further complicates translation, as hyperacute pathological changes and potential therapeutic windows are frequently missed in human imaging [71].

### 4.5. Experimental Clearance-Enhancing Interventions in SAH Models

#### 4.5.1. AQP4 and Glymphatic Modulation

AQP4 channels are critical regulators of glymphatic inflow. After SAH, reactive astrogliosis leads to AQP4 depolarization, which disrupts the directional water flow necessary for effective solute transport. Preclinical studies have shown that genetic deletion of AQP4 impairs glymphatic function. The pharmacological agents that stabilize perivascular AQP4 polarization can restore flow and improve clearance of neurotoxic proteins such as Aβ and tau [70].

#### 4.5.2. Pharmacological and CSF-Dynamics Approaches

Several therapeutic approaches are currently under investigation, including pharmacological agents to reduce oxidative stress and restore normal CSF circulation. Pharmacological agents such as acetazolamide and furosemide have been used to manipulate CSF production and dynamics, though with mixed results [70].

Tissue plasminogen activator (tPA) improved neurological outcomes in mice after 7 days, which was associated with improved glymphatic clearance [73,91]. Nimodipine attenuated early brain injury, including edema and neurological dysfunction, and was associated with improved glymphatic function after SAH in mice [92]. Treatment with pituitary adenylate cyclase-activating polypeptide (PACAP) increased glymphatic function after SAH and alleviated the subsequent increase in ICP and brain water content [93].

In a beagle model, intermittent cistern magna CSF drainage significantly ameliorated glymphatic and meningeal lymphatic dysfunction after SAH [88]. Daily intermittent cistern magna CSF drainage also reduced ventricular dilation and PVS enlargement by restoring glymphatic and meningeal lymphatic functions [88]. Clinically, intraventricular tPA administration and CSF drainage or flushing has been explored to accelerate clot resolution and improve outcomes, although favorable effects on clot reduction have not consistently translated into improved DCI or functional outcomes [70].

#### 4.5.3. Meningeal Lymphatic Enhancement

Meningeal lymphatic vessels are one of the proposed efflux routes, draining interstitial fluid and waste products from the brain to the cervical lymphatic system, although the relative importance of the different routes is still debated [2]. In preclinical studies, vascular endothelial growth factor C (VEGF-C) or CCL21 enhances lymphatic drainage to dCLNs and reduces meningeal T helper 17 (Th17) accumulation. In parallel, early increases in CSF cytokines and coagulation-related factors have been shown to predict the development of chronic hydrocephalus [70]. This supports a role for inflammation–coagulation coupling in impaired CSF circulation and clearance [70].

The close relationship between glymphatic dysfunction and neuroinflammation makes anti-inflammatory therapies an attractive avenue for therapeutic intervention. After SAH, proinflammatory cytokines such as TNF-α, IL-1β, and interleukin-6 (IL-6) have been shown to be elevated [94]. These cytokines play a crucial role in the secondary injury cascades. Modulating neuroinflammation could indirectly improve glymphatic flow by preserving the integrity of perivascular structures and restoring normal vascular compliance [94].

**Figure 3:**
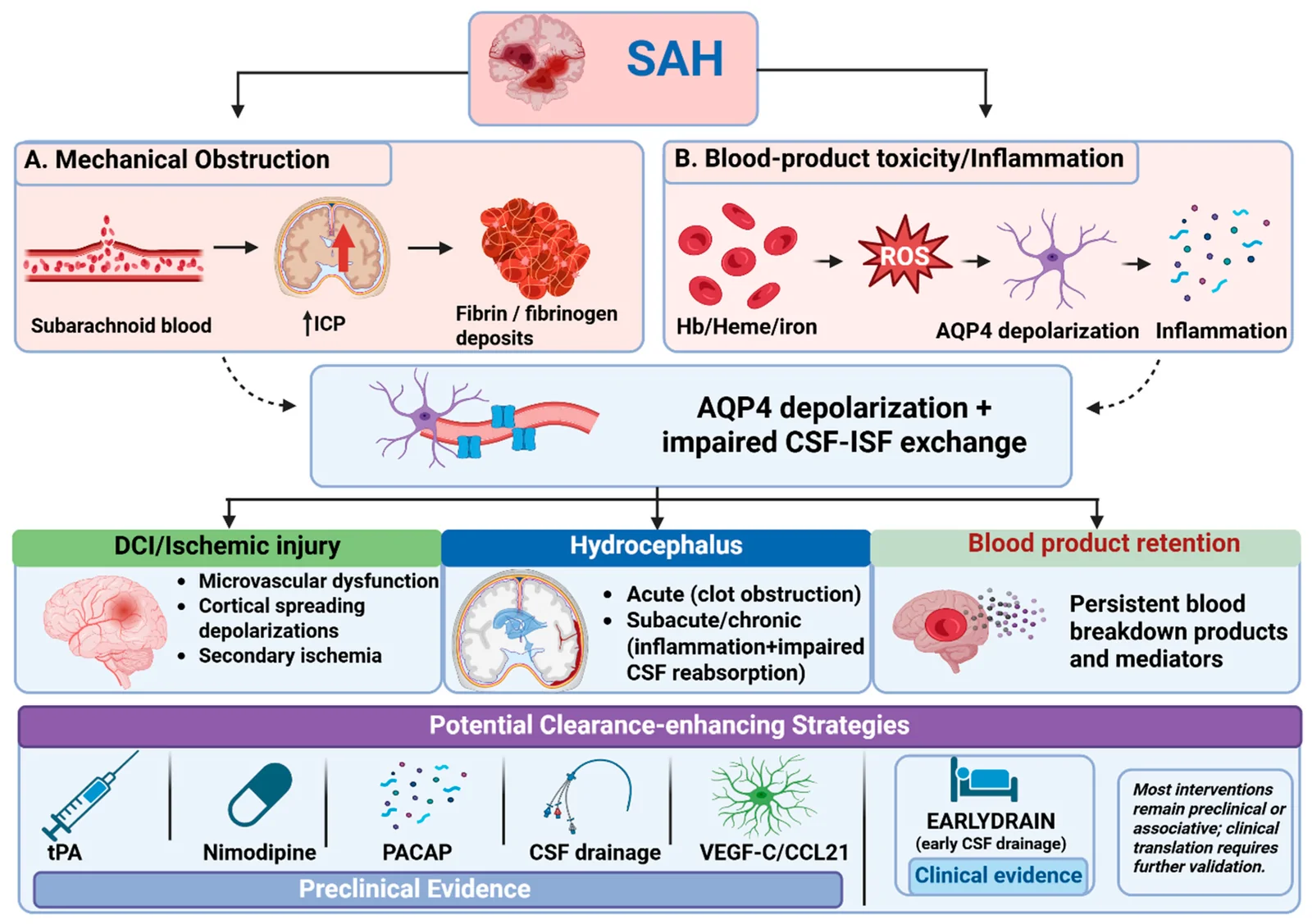
Glymphatic dysfunction and clearance-enhancing strategies in SAH. (**A**) Mechanical obstruction by subarachnoid blood, raised ICP, and fibrin/fibrinogen deposits. (**B**) Blood-product toxicity and inflammation in which Hb, heme, and iron generate ROS, leading to AQP4 depolarization and inflammation. Both pathways converge on impaired CSF–ISF exchange, driving DCI, hydrocephalus, and blood product retention. Preclinical (tPA, nimodipine, PACAP, CSF drainage, VEGF-C/CCL21) and clinically validated (EARLYDRAIN) strategies are shown. **Abbreviations:** AQP4, aquaporin-4; CCL21, C-C motif chemokine ligand 21; CSF, cerebrospinal fluid; DCI, delayed cerebral ischemia; EARLYDRAIN, early lumbar CSF drainage trial; Hb, hemoglobin; ICP, intracranial pressure; ISF, interstitial fluid; PACAP, pituitary adenylate cyclase-activating polypeptide; ROS, reactive oxygen species; SAH, subarachnoid hemorrhage; tPA, tissue plasminogen activator; VEGF-C, vascular endothelial growth factor C.

Overall, targeting glymphatic and lymphatic pathways may mitigate secondary brain injury and attenuate systemic inflammatory responses [70]. Although promising, current evidence remains largely preclinical or associative, and translation into clinical practice requires further human studies. The convergent pathways of glymphatic failure after SAH, along with candidate clearance-enhancing strategies, are illustrated in Figure 3.

## 5. Glymphatic Failure as a Bridge to Neurodegeneration

Impaired glymphatic clearance may result in the retention and accumulation of neurotoxic proteins such as phosphorylated tau and Aβ [1,54]. The relevance of these processes to TBI-related neurodegeneration is discussed in Section 3.5.

Sleep disturbance may link glymphatic dysfunction to protein accumulation. Four studies assessing symptomatic neurotraumatic disorders, ranging from TBI to stroke, found associations between sleep disturbances and increased Aβ deposits and tau protein levels [95]. Three results showed that elevated Aβ deposits were more consistently associated with sleep disorders. Interleukin 8 (IL-8) levels are elevated in association with sleep problems in studies that assessed only interleukins [95].

Impaired glymphatic waste removal has been proposed as one contributor to major neurodegenerative dementias, although its causal role remains unproven [69]. Alzheimer’s disease is currently irreversible, and sleep disruptions are associated with an increased risk of Alzheimer’s disease. Poor sleep quality is associated with accumulation of Alzheimer’s disease-related Aβ and tau pathology [96]. Alzheimer’s disease is characterized by the accumulation of Aβ, and reduced clearance has been proposed as one contributing factor [96,97]. In this clearance pathway, the movement of CSF from the subarachnoid space to the parenchyma is mediated by the astroglial AQP4 [97].

Similarly, glymphatic dysfunction may contribute to Parkinson’s disease pathogenesis. Experimental studies suggest that impaired glymphatic clearance, together with loss of AQP4 polarization, may contribute to the accumulation of extracellular α-synuclein, which is associated with Lewy body pathology and dopaminergic neuronal loss in the substantia nigra [98]. These findings are largely preclinical, and a causal role in human Parkinson’s disease has not been established. Waste removal from the CNS is essential for maintaining homeostasis throughout life [99]. Glymphatic dysfunction has been associated with age-related cognitive decline [100]. Age-related loss of AQP4 polarization and decreased arterial elasticity impair glymphatic efficiency, and the resulting decline in clearance may contribute to age-associated cognitive deterioration [100].

Beyond Aβ and tau, evidence linking glymphatic impairment to other aggregation-prone proteins is considerably more limited. For α-synuclein, experimental data suggest that impaired glymphatic clearance with loss of AQP4 polarization promotes its extracellular accumulation [98], but direct evidence in humans remains lacking. TDP-43 accumulation is observed in CTE (Section 3.5), yet none of the studies reviewed here directly tested whether its clearance depends on glymphatic function, so any link remains speculative. Amyloid precursor protein accumulation after diffuse brain injury was reduced when AQP4 polarization and glymphatic function were preserved [101], suggesting that clearance of other injury-related proteins may also depend on this pathway. Similarly, glymphatic efflux has been proposed to carry TBI-related proteins such as GFAP, S100B, and NSE into the circulation [56]. Whether these observations extend to pathological aggregates other than Aβ and tau requires further study.

## 6. Imaging and Biomarker Approaches to Glymphatic Assessment

Quantitative assessment of glymphatic kinetics in vivo remains technically challenging. However, there are certain methods such as DTI-ALPS index, intrathecal gadolinium-based MRI tracers, and positron emission tomography (PET)-based approaches to estimate glymphatic flow. DTI-ALPS offers a non-invasive surrogate metric of glymphatic function by measuring water diffusivity along deep medullary perivascular spaces relative to orthogonal white matter tracts. The water diffusivity along this direction can reflect glymphatic system function to investigate any pathology in the glymphatic system [102]. However, it is not a direct method to estimate glymphatic flow and measures it indirectly through water diffusivity [103].

Although DTI-ALPS provides a non-invasive approach, it is still indirect in a certain way, as tracer-based techniques such as intrathecal GE-MRI provide a more direct assessment of the glymphatic system. Conversely, intrathecal contrast-enhanced MRI (CE-MRI) using gadolinium-based contrast agents is currently the most direct approach for visualizing glymphatic transport in humans, although it has not been established as a gold standard. The CSF tracer diffuses centripetally from the surrounding structures deep into the brain parenchyma. It is used to diagnose any underlying pathology [104]. CSF tracer-enhanced MRI is a more direct method to assess CSF and glymphatic transport, but it is limited by invasive procedures such as lumbar puncture and serious adverse effects [105].

Despite being a direct method to assess glymphatic transport, CE-MRI is still unsuitable for routine assessment. Positron emission tomography-based approaches are being explored. These approaches use positron emission tomography to see how long it takes to clear a radioactive tracer that was administered from the ventricles [68]. However, there is impaired clearance associated with Aβ protein accumulation, and it remains uncertain which components of brain function are quantifiable using different neuroimaging methods [68]. Although PET can provide quantitative information, biochemical biomarkers in blood and CSF offer a complementary, less invasive approach to examining molecular changes associated with impaired brain clearance. Brain-derived molecules such as GFAP, S100B, and ubiquitin C-terminal hydrolase L1 (UCH-L1) have been used mainly to diagnose CNS or neurological disease. For example, S100B is elevated after brain trauma [106]. Biochemical biomarkers are minimally invasive, but they lack specificity for glymphatic activity.

Despite advances, no currently available method is fully non-invasive and provides a specific measurement of glymphatic flow. There is no gold standard for the assessment of the glymphatic system. The emerging non-contrast MRI methods, miniature fluorescent microscopy, genetic manipulation, and in vitro microfluidic methods are, therefore, essential for establishing reliable biomarkers to assess the glymphatic system [107].

## 7. Therapeutic and Translational Implications

Therapeutic strategies for glymphatic dysfunction are best understood in terms of the mechanisms by which clearance may be restored, which differ between TBI and SAH. In TBI, loss of perivascular AQP4 polarization is a proposed mechanism of glymphatic impairment, so restoration is framed as re-establishing polarization rather than increasing total AQP4 expression [108]. Fingolimod and angiotensin II type 2 receptor activation improved AQP4 polarization and glymphatic function in animal models [101,109]. In SAH, proposed mechanisms include obstruction by blood and fibrin together with raised ICP, so restoration would depend on clearing this material and relieving pressure. This is the rationale for tPA, CSF drainage, nimodipine, and PACAP in experimental models [88,91,92,93] and for early lumbar drainage in the EARLYDRAIN trial [110].

Two routes apply to both conditions: enhancement of meningeal lymphatic drainage, for example with VEGF-C [111,112,113], and sleep-related physiological regulation, in which reduced noradrenergic tone and slow vasomotion promote CSF–ISF exchange [8,23]. Most of this evidence is preclinical, and several interventions also have anti-inflammatory or vascular effects [112]. The individual approaches are discussed below, beginning with sleep optimization.

### 7.1. Sleep Optimization as a Clearance-Enhancing Intervention

Sleep optimization represents a potentially modifiable way to enhance brain fluid and metabolic clearance, because sleep state genuinely influences CSF dynamics and molecular transport rather than merely correlating with them (Section 2.5). Conversely, sleep physiology can be deliberately modified as demonstrated from the interventional studies. Phase-locked acoustic stimulation delivered over multiple nights has been used to enhance slow-wave activity, and the size of that electrophysiological response tracked with favorable Aβ dynamics and better memory in older adults with cognitive impairment [114]. Interventions targeting sleep optimization are also feasible in a TBI population specifically. Randomized trials have shown that morning blue-wavelength light therapy improves sleep timing, daytime sleepiness and executive function after mTBI, while adapted cognitive-behavioral therapy can produce lasting improvements in sleep quality and fatigue [115,116]. Altogether, these findings support sleep optimization as a good option worth trying. This includes restoration of enough sleep duration and timing, treatment of insomnia after injury, resetting the circadian rhythm, and newer methods to augment slow-wave activity for better sleep. It is a low-risk intervention to help set the physiological conditions that facilitate fluid transport in brain and metabolic clearance.

### 7.2. Pharmacological AQP4 Modulation (Experimental Agents)

AQP4 represents a potential therapeutic target for glymphatic dysfunction because its polarized localization at astrocytic endfeet is strongly linked to perivascular fluid transport, even though the exact contribution of AQP4 to glymphatic influx is still being actively investigated [108]. Experimental findings indicate that restoring AQP4 polarization, rather than simply boosting overall AQP4 expression, may offer a more mechanistically sound approach for improving glymphatic-related transport following brain injury [108]. Supporting this idea, a rat model of diffuse brain injury showed that fingolimod treatment reduced the loss of AQP4 polarization, improved tracer-based glymphatic function, reduced both brain edema and amyloid precursor protein buildup, and improved neurological outcomes. This provided preclinical evidence for the notion that pharmacological preservation of AQP4 polarization may aid recovery after diffuse TBI [101]. Importantly, when the glymphatic system was pharmacologically inhibited, some of the beneficial effects of fingolimod were diminished and amyloid precursor protein accumulation increased, supporting the idea that glymphatic restoration contributed to the observed effects. However, the fingolimod also exerts anti-inflammatory effects; its therapeutic benefits cannot be attributed solely to glymphatic modulation [101]. Recently, a study found out that activation of the angiotensin II type 2 receptor with the experimental agonist compound 21 improved both glymphatic influx and clearance in mice following TBI. This was accompanied by restoration of perivascular AQP4 polarization, dampened neuroinflammatory response, reduced Aβ and phosphorylated tau accumulation, and improvement in both motor function and cognition [109]. Collectively, these results provide proof of concept that alteration of pathways involved in AQP4 polarization and glymphatic transport can modify post-traumatic pathology in animal models, though they stop short of confirming that AQP4-directed therapy would be effective in human TBI patients [101]. For these reasons, pharmacological strategies aimed at AQP4 should, for now, be regarded as a promising preclinical therapeutic strategy. Additional studies will be necessary to determine whether restoration of AQP4 polarization can reliably enhance glymphatic function and, more importantly, whether such improvements translate into genuine, clinically meaningful neurological outcomes in humans [108].

### 7.3. Meningeal Lymphatic-Targeted Approaches (VEGF-C Augmentation, Preclinical)

Meningeal lymphatic vessels also appear to play a role in this broader clearance system. Studies have shown that impairment in the meningeal lymphatic function reduces the clearance of macromolecules from the brain. Conversely, VEGF-C treatment in aged mice to enhance meningeal lymphatic drainage improved learning/memory [111]. Similar benefits have been observed in rat models, where VEGF-C and other lymphatic-modulating interventions improved meningeal lymphatic function, promoted CSF drainage and edema absorption, reduced neuroinflammation and improved neurological outcomes [112]. Experimental studies have provided proof of concept that VEGF-C can enhance this drainage pathway. In aged mice, VEGF-C treatment increased meningeal lymphatic drainage of CSF macromolecules and was associated with improved brain perfusion and cognitive performance [111]. This concept has subsequently been extended to TBI, in which TBI produced persistent impairment of meningeal lymphatic drainage, and VEGF-C-mediated restoration of lymphatic function reduced gliosis in aged mice [113].

### 7.4. Posture/CSF Dynamics Interventions Post-SAH

Interventions that target CSF dynamics represent a complementary route for speeding the removal of blood and its degradation products from the subarachnoid compartment after SAH. Experimental and computational modeling demonstrates that body orientation and lumbar drainage rate influence the efficiency of cranial blood clearance. Upright position in particular appeared to maximize diversion in an anatomically based bench-top model. However, this postural effect has not yet been validated as a clinical intervention in patients with SAH [117]. Human phase-contrast MRI work further shows that changing body position measurably alters CSF exchange between the cranial and cervical compartments [118]. This provides a physiological support for the concept that posture can modify CSF dynamics, although this evidence was obtained from healthy volunteers rather than patients with SAH. Stronger clinical evidence comes from the EARLYDRAIN trial, which was a randomized multicenter trial study. It demonstrated that early controlled lumbar CSF drainage after the treatment of aneurysm reduced both secondary infarction and unfavorable neurological outcomes at six months [110]. Follow-up analysis of the same EARLYDRAIN cohort suggests that drainage volume and intracranial-pressure dynamics can both influence the outcome. This emphasizes the importance of optimizing rather than simply maximizing CSF removal [119]. The biological rationale for this approach is reinforced by observational evidence linking faster clearance of CSF red blood cells and total protein with better functional outcome after SAH [120]. These findings suggest controlled manipulation of CSF dynamics as a plausible way to facilitate the removal of hemorrhage byproducts after SAH, although optimal drainage rate, duration, patient positioning and patient selection remain to be established.

### 7.5. Barriers to Clinical Translation and Trial Design Considerations

Several barriers currently limit translation of glymphatic targeted therapies into clinical practice. Firstly, there is no single non-invasive measure that has been validated as a definitive pharmacodynamic marker of human glymphatic function. DTI-ALPS values are altered following TBI and have been associated with injury severity and clinical symptoms [121]. This is an important gap, underscoring the need for biological validation before ALPS can be used as a surrogate endpoint in therapeutic trials. Secondly, DTI-ALPS is not measured the same way from one research center to another; regions of interest differ and so do the scanner characteristics. This kind of variability may compromise reproducibility across centers, and importantly, future multicenter studies would require prespecified acquisition protocols, harmonized image-processing pipelines and careful quality control [122]. Thirdly, glymphatic changes seem not to be static following TBI; they evolve over time, and their magnitude may vary with injury severity, diffuse axonal injury, SAH and other clinical features. Therapeutic trials should take into account the longitudinal assessment and stratification of patients according to injury severity and timing, as TBI is not a biologically uniform condition [59]. Finally, many preclinical interventions that are aimed to enhance AQP4 polarization or meningeal lymphatic function also happen to have additional anti-inflammatory, vascular or edema-modifying effects. This overlap makes it difficult to state that any functional recovery is specifically because of restoration of glymphatic clearance rather than these other effects [112]. This also applies to gasotransmitter-based approaches: NaHS [49,50] and NOS inhibition [51] reduced brain water content, and NaHS reduced neuroinflammation [49] and oxidative-stress markers [50], so the benefit cannot be attributed to glymphatic restoration without direct clearance measurement. Table 1 summarizes the level of evidence (animal, human, and clinical intervention) for the main topics discussed in Section 3, Section 4, Section 5, Section 6 and Section 7 and indicates which findings are better established and which remain preliminary.

## 8. Gaps in Current Knowledge and Future Directions

### 8.1. Lack of Standardized Human Glymphatic Imaging Protocol

Several imaging studies point toward glymphatic impairment after TBI, but each comes with real limitations that are worth keeping in view. A study comparing 89 TBI patients with 34 healthy controls found that the DTI-ALPS index was lower in the injured group, and the lower scores were associated with injury severity and the presence of SAH/diffuse axonal injury [121]. A separate prospective study evaluated 44 patients with mTBI at approximately 14 days and 6–12 months post-injury and illustrates exactly why longitudinal assessment is needed in this field rather than relying on single time snapshots [59]. On the mechanistic side, intrathecal gadolinium-enhanced MRI demonstrated organized CSF movement through perivascular spaces into the brain parenchyma in humans, genuine evidence that this physiological pathway exists. That being said, this should not be oversold as a validated clinical test of glymphatic function. It was a proof-of-principle study in only five patients, a limitation the NIH’s own summary of the work also flags [125]. Human studies are increasingly building support for perivascular CSF transport as a real phenomenon, but the available techniques interrogate different components of the glymphatic–meningeal lymphatic axis and are still limited by invasive methods, indirect measurements, inconsistent methodology and lack of a standardized way to acquire and analyze this kind of data.

### 8.2. Need for Longitudinal Cohort Data Linking Acute Injury to Long-Term Glymphatic/Cognitive Outcomes

Emerging human studies reveal that abnormalities related to the glymphatic system may accompany TBI and relate to continuous symptoms, but their longitudinal evidence remains limited. Research in mTBI has demonstrated that measurement of perivascular-space burden and DTI-ALPS in the acute phase helps in prediction of chronic memory problems, while serial ALPS measurements have shown how the change in these values over time may track symptom progression more closely than a single baseline reading [59,126]. Studies of chronic TBI have additionally linked lower ALPS values with cognitive impairment, but because these are cross-sectional observations, they cannot determine whether glymphatic dysfunction comes first or only accompanies chronic cognitive deficits [123]. Existing human studies indicate that abnormalities related to glymphatics following a TBI are dynamic and may relate to persistent symptoms and cognition, but available cohorts are small, predominantly focused on mTBI, and generally have limited follow-up. Therefore, properly powered longitudinal cohorts are needed that follow patients from the acute through the subacute and chronic stages of TBI so that the researchers can determine whether early changes in biomarkers related to the glymphatic system can predict long-term cognitive trajectories and whether the ongoing dysfunction plays a causal role in post-traumatic neurodegeneration.

### 8.3. Species-Translation Gap (Most Tracer Data Remains Rodent-Derived)

A second major gap concerns how well the mechanistic tracer findings translate across species. Peters & Lyketsos made this point by directly stating that much of the literature related to the glymphatic system in TBI rests on mouse models, and replicating it in humans remains an unresolved challenge [60].

Studies of the perivascular system have shown that macromolecules can be transported along perivascular pathways within the CNS, highlighting the importance of these pathways in brain fluid and solute transport [127]. In addition, studies in rhesus and cynomolgus macaques have shown that macromolecules can move through perivascular pathways and ultimately drain from the skull through cervical lymphatic channels [128]. Human and non-human primates’ meningeal lymphatics can also be visualized using clinical MRI, suggesting this lymphatic architecture is substantially conserved across species [19].

The available large-animal and primate datasets remain comparatively small, and we still lack solid, quantitative comparisons across species in terms of tracer kinetics, perivascular transport and clearance mechanisms. Hence, future studies should make more use of the gyrencephalic large-animal and non-human primate models alongside human imaging to determine which aspects of rodent glymphatic physiology hold up in more human-like brains and which depend on species-specific factors like neuroanatomy, brain size, gyrification pattern and CSF dynamics.

### 8.4. Opportunity for a Prospective TBI/SAH Cohort with Serial DTI-ALPS and Cognitive Follow-Up

These gaps point toward a clear opportunity for a prospective longitudinal cohort study following TBI and SAH patients over time and incorporating repeated DTI-ALPS scans along with structural MRI and standardized cognitive testing. Prospective human studies have shown that combining DTI-ALPS with cognitive testing after mTBI is feasible, while longitudinal studies have shown that ALPS values can be measured over months and may help track how well the patients ultimately recover [35,59,126]. Likewise, in SAH patients, prospective tracer-based MRI has shown that glymphatic function can be substantially and variably impaired depending on when it is measured after bleed. This makes a strong point for serial rather than relying on single-time-point assessment [124].

Building on this, a unified cohort design could, therefore, assess participants during the acute injury phase and then follow them at predefined subacute and chronic stages. This would allow researchers to investigate whether early glymphatic abnormalities predict later cognitive decline, persistent post-concussion symptoms, hydrocephalus or broader functional outcomes.

## 9. Conclusions

The glymphatic system is a multi-tiered clearance network whose function depends on the coordinated interaction of astrocytic AQP4 polarization, meningeal lymphatic drainage, arterial pulsatility, and sleep-, posture-, and circadian-dependent regulation. Aging impairs several of these components at once, which may compromise clearance even when individual mechanisms remain partly preserved.

In TBI, mechanical shear and NLRP3-dependent inflammatory signaling may disrupt perivascular AQP4 polarization, and glymphatic impairment has been reported from 1 day to at least one month after injury in rodent models, although AQP4 expression and polarization patterns differ between clinical and experimental studies. The resulting reduction in solute clearance may promote tau and Aβ accumulation, which may represent one candidate mechanism linking TBI to CTE and other chronic neurodegenerative consequences, although causality has not been established. In SAH, blood and fibrin obstruction of perivascular pathways, hemoglobin- and iron-related toxicity, and AQP4 depolarization suppress CSF–ISF exchange, and clearance failure appears to be linked to DCI, SDs, and post-hemorrhagic hydrocephalus.

Reduced clearance of Aβ, phosphorylated tau, and α-synuclein may contribute to Alzheimer’s and Parkinson’s disease, and sleep disturbance appears to link injury-related glymphatic impairment with protein accumulation.

Several imaging and biomarker strategies have been proposed to assess glymphatic function, including DTI-ALPS, intrathecal gadolinium MRI, PET-based tracer clearance, and fluid biomarkers. However, each of these captures only part of the underlying process, and none has emerged as a validated gold-standard measure. The lack of a reliable pharmacodynamic marker, together with variability in DTI-ALPS acquisition across centers and time-dependent changes after injury, remains a major barrier to clinical translation.

From a therapeutic perspective, sleep optimization is one of the most practical and low-risk approaches. Evidence from multiple studies suggests that sleep actively promotes glymphatic clearance rather than simply being associated with it. Pharmacological strategies that target AQP4 or enhance meningeal lymphatic function through VEGF-C have shown encouraging results in preclinical studies. Further validation is required to determine whether the benefits of AQP4-directed strategies are specifically mediated through glymphatic restoration or from concurrent anti-inflammatory and vascular effects. Among interventions that target CSF dynamics, controlled lumbar drainage has the strongest clinical evidence. The EARLYDRAIN trial showed improved six-month outcomes with early drainage in SAH patients, while postural approaches remain limited to experimental and healthy-volunteer data.

Overall, these findings suggest that the glymphatic–meningeal axis may be one pathway linking acute neurotraumatic injury and chronic neurodegeneration alongside other contributing mechanisms. Translating these observations into clinical practice will require standardized and reproducible imaging protocols, sufficiently powered longitudinal studies covering acute and chronic stages of injury, and expanded investigation in gyrencephalic large-animal and non-human primate models. These approaches are required to clarify which mechanisms identified in rodents are applicable to humans. A prospective cohort with serial DTI-ALPS imaging and standardized cognitive follow-up in TBI and SAH represents a feasible next step for evaluating glymphatic integrity as a potential marker of injury severity and future neurodegenerative risk.

## Figures and Tables

**Table 1:** Evidence-level summary of key glymphatic topics in traumatic brain injury, subarachnoid hemorrhage, and sleep, separating animal evidence, human evidence, and clinical intervention evidence.

Topic	Animal Evidence	Human Evidence	Clinical Intervention Evidence	Overall Status
**AQP4 disruption after TBI**	Perivascular AQP4 polarization disrupted in mild/moderate TBI models; AQP4 KO worsens glymphatic dysfunction, p-tau, cognitive decline [44,56]; NLRP3 link [40]	Clinical studies mostly show AQP4 upregulation (CSF/plasma), discordant with experimental patterns [52,66]; polarization not directly measured	None in humans; fingolimod and compound 21 are preclinical only [101,109]	Preliminary
**Reduced glymphatic clearance after TBI**	~60% reduced CSF influx, persists > 28 days [54]; CE-MRI influx/efflux deficits at 1 day to 1 month [55]; regional variation after repetitive mTBI [57]	Indirect only; both increases and decreases reported [63]; enlarged PVS on MRI [61]	None	Strong in rodents, inconsistent in humans
**DTI-ALPS after TBI**	Not applicable (human imaging index)	Lower ALPS in 89 TBI vs. 34 controls, linked to severity [121]; mTBI longitudinal cohort (n = 44) [59]; NfL association [38]; cross-sectional cognitive link [123]	None; not validated as surrogate endpoint [121]	Associative, not validated
**Glymphatic dysfunction after SAH**	Beagle CE-MRI: reduced within 1 h, persists 1 week, partial recovery 2 weeks [70,88]; impaired tracer distribution in rodents and NHP [73]; fibrin occlusion [71,74]	Erythrocytes in PVS postmortem [71]; prospective tracer MRI shows variable, time-dependent impairment [124]	tPA, nimodipine, PACAP preclinical [91,92,93]; intraventricular tPA/flushing clinically inconsistent [70]	Moderate in animals, limited in humans
**Sleep optimization**	Sleep gives ~60% ISF expansion and ~2× clearance [8]; posture [9], circadian [10] effects	EEG-fMRI-CSF coupling in NREM [11]; sleep deprivation impairs tracer clearance [24]; sleep disturbance linked to Aβ/tau in neurotrauma [95]	Acoustic stimulation [114], blue light and CBT in mTBI [115,116] improve sleep outcomes	Mechanism moderate, glymphatic benefit unproven
**Lumbar drainage after SAH**	Intermittent cisterna magna drainage in beagles [88]	Faster CSF RBC/protein clearance linked to better outcome [120]	EARLYDRAIN RCT: fewer secondary infarctions and unfavorable outcomes at 6 months [110,119]	Strongest clinical evidence

Abbreviations: Aβ, amyloid-β; ALPS, analysis along the perivascular space; AQP4, aquaporin-4; CBT, cognitive–behavioral therapy; CE-MRI, contrast-enhanced magnetic resonance imaging; CSF, cerebrospinal fluid; DTI-ALPS, diffusion tensor imaging analysis along the perivascular space; EARLYDRAIN, early lumbar CSF drainage trial; EEG, electroencephalography; fMRI, functional magnetic resonance imaging; ISF, interstitial fluid; KO, knockout; mTBI, mild traumatic brain injury; NfL, neurofilament light chain; NHP, non-human primate; NLRP3, NLR family pyrin domain-containing 3; NREM, non-rapid eye movement; PACAP, pituitary adenylate cyclase-activating polypeptide; p-tau, phosphorylated tau; PVS, perivascular space; RBC, red blood cell; RCT, randomized controlled trial; SAH, subarachnoid hemorrhage; TBI, traumatic brain injury; tPA, tissue plasminogen activator.

## Data Availability

No new data were created or analyzed in this study. Data sharing is not applicable to this article.

## Abbreviations

Aβ	amyloid-β
ALPS	analysis along the perivascular space
APP	amyloid precursor protein
APP/PS1	amyloid precursor protein/presenilin 1
AQP4	aquaporin-4
AQP4X	aquaporin-4 readthrough isoform
BBB	blood–brain barrier
BOLD	blood-oxygen-level-dependent
CaM	calmodulin
CBT	cognitive–behavioral therapy
CCL21	C-C motif chemokine ligand 21
CE-MRI	contrast-enhanced magnetic resonance imaging
CNS	central nervous system
COUP-TFII	chicken ovalbumin upstream promoter transcription factor II
CSF	cerebrospinal fluid
CTE	chronic traumatic encephalopathy
DCI	delayed cerebral ischemia
dCLNs	deep cervical lymph nodes
DTI	diffusion tensor imaging
DTI-ALPS	diffusion tensor imaging analysis along the perivascular space
EARLYDRAIN	early lumbar CSF drainage trial
EEG	electroencephalography
EVD	external ventricular drainage
FLAIR	fluid-attenuated inversion recovery
fMRI	functional magnetic resonance imaging
GCS	Glasgow Coma Scale
Gd-DTPA	gadolinium diethylenetriamine penta-acetic acid
GFAP	glial fibrillary acidic protein
H_2_S	hydrogen sulfide
Hb	hemoglobin
ICP	intracranial pressure
IL-1β/IL-6/IL-8	interleukin-1β/-6/-8
iNOS	inducible nitric oxide synthase
ISF	interstitial fluid
LRP1	low-density lipoprotein receptor-related protein 1
LYVE-1	lymphatic vessel endothelial hyaluronan receptor 1
MCAO	middle cerebral artery occlusion
MMP/MMPs	matrix metalloproteinase(s)
mTBI	mild traumatic brain injury
MRI	magnetic resonance imaging
NAC	N-acetylcysteine
NaHS	sodium hydrosulfide
NfL	neurofilament light chain
NHP	non-human primate
NLRP3	NLR family pyrin domain-containing 3
NMDA	N-methyl-D-aspartate
NO	nitric oxide
NOS	nitric oxide synthase
NREM	non-rapid eye movement
NSE	neuron-specific enolase
OAPs	orthogonal arrays of particles
PACAP	pituitary adenylate cyclase-activating polypeptide
PET	positron emission tomography
PKA	protein kinase A
p-tau	phosphorylated tau
PROX1	prospero homeobox protein 1
PVS	perivascular space
RBC	red blood cell
RCT	randomized controlled trial
ROS	reactive oxygen species
S100B	S100 calcium-binding protein B
SAH	subarachnoid hemorrhage
SD/SDs	cortical spreading depolarization(s)
TBI	traumatic brain injury
TDP-43	transactive response DNA-binding protein 43
Th17	T helper 17
TIMP	tissue inhibitor of metalloproteinases
TNF-α	tumor necrosis factor-α
tPA	tissue plasminogen activator
TRPV4	transient receptor potential vanilloid 4
UCH-L1	ubiquitin C-terminal hydrolase L1
VEGF-C	vascular endothelial growth factor C
VEGFR3	vascular endothelial growth factor receptor 3
ZO-1	zonula occludens-1
